# The efficacy of antifibrinolytic therapy in aneurysmal subarachnoid hemorrhage: a systematic review and meta-analysis

**DOI:** 10.2144/fsoa-2023-0014

**Published:** 2023-05-16

**Authors:** Kaneez Fatima, Muhammad Aemaz Ur Rehman, Abyaz Asmar, Hareem Farooq, Noor-Us-Sabah Ahmad, Ahmad Danial, Muhammad Ebaad Ur Rehman, Abdullah Ali Khan, Sidra Tahir, Umair Ahmed, Salman Zubair, Ayaz Khawaja

**Affiliations:** 1 Dow University of Health Sciences, Mission Rd, New Labour Colony Nanakwara, Karachi, Sindh, 74200, Pakistan; 2 Massachusetts General Hospital, 55 Fruit Street Boston, MA 02114, USA; 3 Mayo Hospital, King Edward Medical University, Neela Gumbad Chowk Anarkali, Lahore, 54000, Pakistan,; 4 Quaid-e-Azam Medical College, Bahawalpur, 63100, Pakistan; 5 Allama Iqbal Medical College, Allama Shabbir Ahmed Usmani Road, Lahore, Punjab, 54550, Pakistan; 6 St Anthony Hospital, 1000 N Lee Ave, Oklahoma City, OK 73102, USA; 7 Wayne State University, 540 E Canfield St, Detroit, MI 48201, USA; ^*^Author for correspondence: aemaz100@gmail.com

**Keywords:** aneurysm, antifibrinolytics, clinical outcome, delayed cerebral ischemia, intracranial hemorrhage, meta-analysis, mortality, rebleeding, subarachnoid hemorrhage, tranexamic acid

## Abstract

**Aim:**

The efficacy of antifibrinolytics in subarachnoid hemorrhage remains unclear due to conflicting evidence from studies.

**Materials & methods:**

Online databases were queried to include randomized controlled trials and propensity matched observational studies. We used Review Manager for the statistical analysis, presenting results as odds ratios with 95% CI.

**Results:**

The 12 shortlisted studies included 3359 patients, of which 1550 (46%) were in the intervention (tranexamic acid) group and 1809 (54%) in the control group. Antifibrinolytic therapy significantly reduced the risk of rebleeding (OR: 0.55; 95% CI: 0.40–0.75; p = 0.0002) with no significant decrease in poor clinical outcome (OR: 1.02; 95% CI: 0.86–1.20; p = 0.85) and all-cause mortality (OR: 0.92; CI: 0.72–1.17; p = 0.50).

**Conclusion:**

In patients with subarachnoid hemorrhage, antifibrinolytics reduce the risk of rebleeding without significantly affecting mortality or clinical outcomes.

Subarachnoid hemorrhage (SAH) accounts for approximately ten percent of all hemorrhagic strokes, with a global incidence of aneurysmal SAH of 7.9 per 100,000 person years [1]. Aneurysmal SAH, the most common cause of SAH, is secondary to the rupture of a saccular aneurysm of one of the cerebral vessels. It occurs most frequently in younger individuals with half the patients less than 50 years of age and is responsible for significant morbidity and mortality [2]. About one to two third of patients die after hemorrhage [3], and about one in ten survivors remain unable to lead an independent life [4].

Rebleeding, a common complication of SAH, is associated with increased mortality, brain death, poor outcomes and complications such as hydrocephalus and respiratory failure [5]. Endovascular or neurosurgical obliteration of the aneurysm can prevent rebleeding. However, the risk (4–20%) is maximal within the first 24 h [6], a window during which many patients are beyond the reach of centers that can provide timely intervention [7]. Antifibrinolytic agents, such as aminocaproic acid and tranexamic acid (TXA), inhibit fibrinolysis of blood clots, and can potentially reduce the risk of rebleeding after a SAH. However, the use of antifibrinolytics in SAH has been limited due to concerns that it may increase the risk of cerebral ischemia – a common complication of SAH that arises 3–14 days after the event. It was thus proposed that short-term (i.e., continued no longer than 3 days) treatment with antifibrinolytics; before the cerebral ischemia window, may improve outcomes without increasing cerebral ischemia [6].

The most recent review by Cochrane in 2013 [2] included only a single study evaluating short-term antifibrinolytic treatment in patients with aneurysmal SAH. It showed promising yet inconclusive results on short-term TXA treatment and hypothesized that new studies exploring the use of early or ultra-early short-term TXA therapy might yield positive results [2]. Since then, further studies evaluating short-term treatment have been published. These newer studies have a sample size larger than all the previous trials combined; have higher methodological rigor and are conducted in an era where the management of SAH is significantly different from the past practices. In the present meta-analysis, our aim was to incorporate the new studies to evaluate the potential benefit of antifibrinolytic therapy in terms of the effects on rebleeding risk, poor clinical outcome, delayed cerebral ischemia and all-cause mortality. By grouping studies based on the duration of TXA use, we specifically sought to determine if short-term versus long-term treatment shows evidence of clinical benefit in SAH. We also decided to group studies on the basis of antivasospasm treatments, seeking to know whether cerebral ischemia prevention offers any additional benefit.

## Materials & methods

This systematic review and meta-analysis is compliant with the Preferred Reporting Items for Systematic review and Meta-Analyses (PRISMA) guidelines and has been registered with The International Prospective Register of Systematic Reviews (PROSPERO ID: CRD42022301879).

### Data sources & search strategy

We searched a total of four electronic databases, namely PubMed/MEDLINE, Cochrane CENTRAL, Science Direct and Google Scholar from their inception to April 2023, and no filters were used for language or study design. We used a combination of the keywords and MeSH terms to generate our search string for PUBMED: (‘subarachnoid hemorrhage’ [MeSH terms] or ‘aneurysmal subarachnoid hemorrhage’ [all fields]) and (‘antifibrinolytic agents’ [MeSH terms] or ‘tranexamic acid’ [MeSH terms] or ‘aminocaproic acid’ [all fields] or ‘antifibrinolytics’ [all fields]). Additionally, the bibliographies of the included studies and previous meta-analyses were manually screened to identify all relevant articles. The details of the complete search strategy are given in Supplementary File 1.

### Study selection

Published randomized controlled trials with a follow-up duration of at least 3 months for clinical outcome assessment or observational studies with adjusted or propensity matched data were selected for this meta-analysis. The included population consisted of adult patients (>18 years) presenting within the first 72 h of aneurysmal SAH. Patients were divided into intervention and control groups. The intervention group received antifibrinolytics (oral or intravenous), such as TXA and Epsilon aminocaproic acid, while the control group consisted of patients receiving placebo/standard of care. Only those studies that reported at least one of the four outcomes (i.e., mortality, poor clinical outcome, rebleeding and delayed cerebral ischemia) were included in our article. Studies that did not meet our inclusion criteria were excluded and only those observational studies were considered which had propensity matched data or provided adjusted odds ratios (OR).

### Data extraction

All the articles retrieved from the literature search were exported to Mendeley software where duplicates were screened for and removed. The remaining articles were carefully assessed by two independent reviewers (Hareem Farooq and Muhammad Ebaad Ur Rehman), and articles were assessed using their titles and abstracts first, and all relevant articles were selected. Full articles were then reviewed and those that met the inclusion criteria were finalized for our study. A third investigator (Muhammad Aemaz Ur Rehman) was consulted to resolve any discrepancies that arose during the entire process. The authors then extracted data related to study design, the number of participants in the intervention and control group, details of the intervention (including drug name, dose, duration and route), ischemia prevention strategies, follow-up time and outcomes. Duration of therapy was divided into two categories, short-term (continued less than 72 h after SAH occurrence) and long-term (continued more than 72 h after SAH). We also grouped studies according to the presence or absence of ischemia prevention (defined as the use of calcium channel antagonists and/or hypervolemic therapy), hence four major subgroups of studies were identified: short-term therapy and medical prevention of ischemia; short-term therapy and no medical prevention of ischemia; long-term therapy and medical prevention of ischemia; long-term therapy and no medical prevention of ischemia.

### Outcomes of interest

The four outcomes extracted from all the studies included: all-cause mortality, rebleeding, poor outcome and cerebral ischemia. Our primary outcome was poor clinical outcome assessed at a follow-up of 3–6 months and defined as death, severe disability, a vegetative state corresponding to a score of 4, 5 or 6 on the modified Rankin scale) or a score of 1, 2 or 3 on the Glasgow outcome scale. Among secondary outcomes, our study pooled data for the following: rebleeding, all-cause mortality and delayed cerebral ischemia (or simply, cerebral ischemia). All-cause mortality encompassed death due to any cause at a minimum of a 3-week follow-up. Due to the variability in the definition of cerebral ischemia among studies, we defined the term cerebral ischemia broadly in our study to mean the presence of any form of ischemia or infarction in the brain, after the SAH, seen on radiographic imaging or determined through clinical features.

### Quality assessment of included studies

The Cochrane risk-of-bias tool [8] was used for the quality assessment of randomized controlled trials while the Newcastle Ottawa Quality Assessment scale [9] was used for the observational study. The risk-of-bias tool assessed all studies in the following domains: randomization, allocation concealment, blinding of participants and personnel, blinding of outcome assessment, detection bias, attrition bias, bias in reporting and other biases. All domains were categorized as ‘high risk of bias’, ‘low risk of bias’ and ‘unclear risk of bias’. The Newcastle Ottawa Scale scored the observational study on the following three parameters: selection, comparability and outcome which were further divided into subcategories. The scale rates studies with a score of 7–9 as high quality, while those with a score of 4–6 are considered high risk and 0–3 as very high risk of having bias.

### Statistical analysis

We conducted our statistical analysis using Review Manager (RevMan) (Computer program) version 5.4. The Cochrane Collaboration. Copenhagen: The Nordic Cochrane Center, 2020. The results from all studies were presented as ORs with 95% CI, were pooled using a Mantel–Haenszel random-effects model and forest plots were made for a graphical representation of the pooling. To assess for subgroup differences, the χ^2^ test was used. Heterogeneity across studies was evaluated using Higgins I^2^ and a value less than 50% for I^2^ was considered acceptable. Begg’s test and a visual inspection of the funnel plot were conducted to evaluate for publication bias. A p-value of less than 0.05 was considered significant in all cases.

## Results

### Literature search

**Figure 1.**
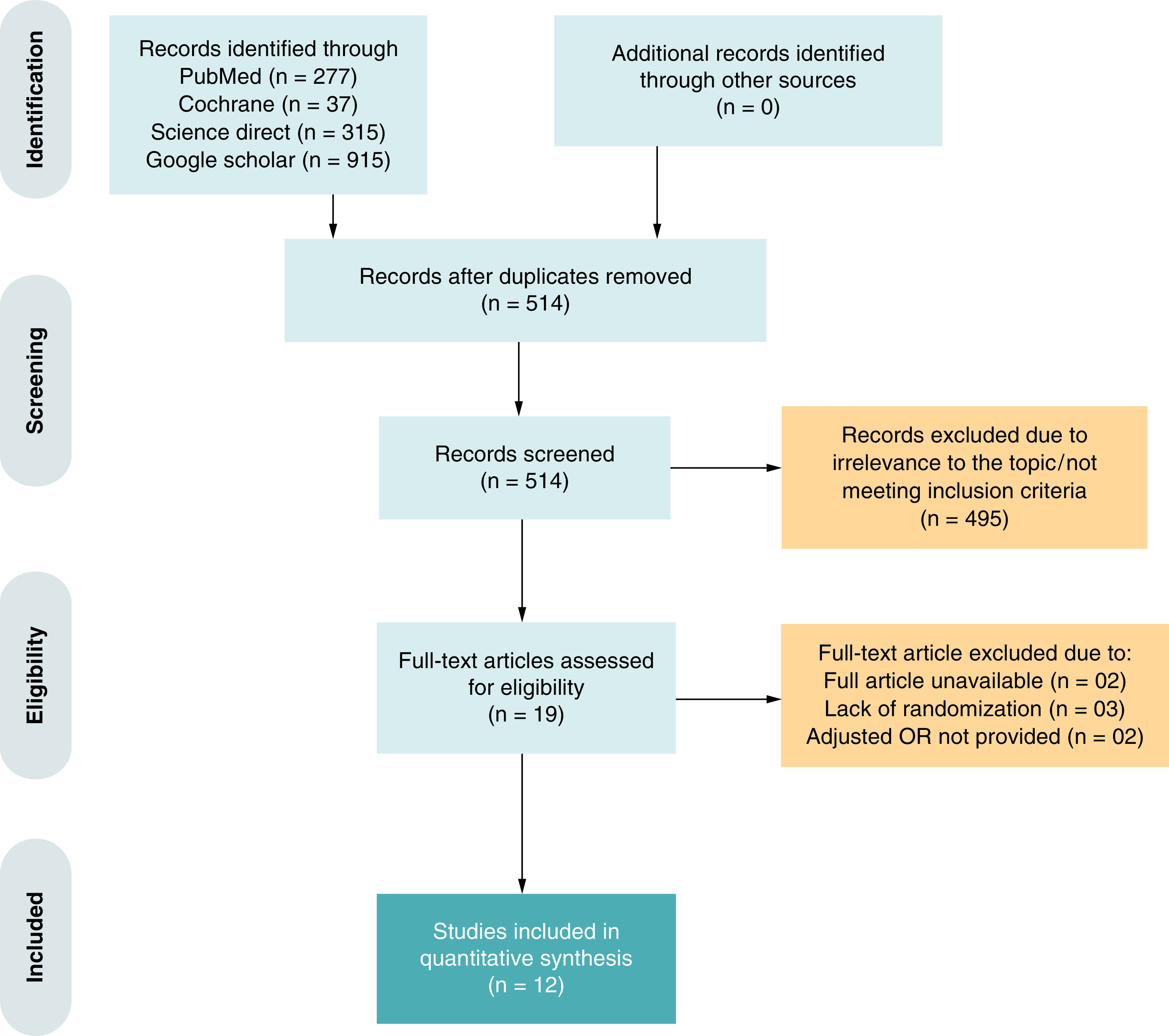
Prisma flow diagram. OR: Odds ratio.

An initial electronic search of four databases yielded 1467 potential studies. Twelve studies were finally selected for analysis, including 11 randomized controlled trials [10–20] and one observational study [21]. The PRISMA flowchart (Figure 1) summarizes the results of our literature search.

### Study characteristics & quality assessment

These studies assigned randomly a total of 3359 patients with SAH: 1550 patients (46%) in the antifibrinolytic treatment arm and 1809 patients (54%) in the control arm. TXA administered either orally or intravenously, was used as an antifibrinolytic in all 12 studies. The follow-up duration for the assessment of clinical outcome ranged from 3 to 6 months. Nine studies belonged to the long-term TXA therapy cohort whereas three studies were placed in the short-term TXA therapy cohort. The baseline study and major intervention characteristics are outlined in Table 1.

**Table 1.** Baseline study and major intervention characteristics of the included studies.

Study (year)/study design	Total (Control [n]/treatment [n])	Intervention (Tranexamic acid)		Ischemic prevention	Major outcomes	Follow-up time for clinical outcome assessment
		Oral/iv. (Dose)	Duration (Short term vs long term)			
Chandra *et al.* 1978/RCT	39 (19/20)	iv. (1 g every 4 h)	14–21 days (long-term)	No	Rebleeding, mortality	Clinical outcome not assessed
Fodstad *et al.* 1981/RCT	59 (29/30)	Both (1 g/100 ml iv. every 4 h for 1 week, then every 6 h in 2nd week and 1.5 g orally every 6 h in week 3–6)	≤6 weeks, discharge, surgery or death (long-term)	No	Mortality, rebleeding, delayed cerebral ischemia, thrombotic complications	Clinical outcome not assessed
Gelmers *et al.* 1980/RCT	57 (26/31)	Both (1 g every 6 h, oral dose not mentioned)	Until surgery or discharge; mean 17 days (long-term)	No	Rebleeding, mortality	Clinical outcome not assessed
Hillman *et al.* 2002/RCT	505 (251/254)	iv. (1 g iv. stat before transporting to surgery followed by 2nd dose of 1 g after 2 h and then continued with 1 g every 6 h up to 72 h)	≤72 h (short-term)	Yes (Calcium antagonists, hypervolemia)	Vasospasm, delayed cerebral Ischemia, rebleeding, clinical outcome (GOS)	6 months
Kaste *et al.* 1979/RCT	64 (32/32)	iv. (1 g every 4 h)	Until surgery or ≤21 days if surgery was not feasible (long-term)	No	Mortality, rebleeding, vasospasm	Clinical outcome not assessed
Maurice Williams. *et al.* 1978/RCT	79 (41/38)	iv. initially then oral (6 g daily)	6 weeks or until operation (long-term)	No	Rebleeding, mortality	Clinical outcome not assessed
Roos *et al.* 2000/RCT	462 (233/229)	iv. (week 1) and oral (weeks 2 and 3)/(6 g daily)	3 weeks (long-term)	Yes (Calcium antagonists, hypervolemia)	Clinical outcome (GOS), rebleeding, delayed cerebral ischemia, hydrocephalus	3 months
Tsementzis *et al.* 1990/RCT	100 (50/50)	iv. initially then oral (9 g daily - iv. in 6 doses in week 1, orally in 4 doses in week 2–4)	Until the time of surgery or 4 weeks from ictus, whichever was sooner (long-term)	No	Rebleeding, delayed cerebral Ischemia, hydrocephalus, clinical outcome (GOS)	6 Months
van Rossum *et al.* 1977/RCT	51 (25/26)	iv. (4 g daily)	10 days or until surgery (long-term)	No	Mortality, rebleeding	Clinical outcome not assessed
Vermeulen *et al.* 1984/RCT	479 (238/241)	Both (6 g daily in 6 doses in week 1 and 4 g daily in 4 doses thereafter)	≤4 weeks or until surgery or any other established diagnosis (long-term)	No	Mortality, rebleeding, cerebral infarction, hydrocephalus clinical outcome (GOS)	3 months
Post *et al.* 2019/Observational cohort study	509 (390/119)	iv. (1 g loading dose on admission to ER or ICU, followed by 1 g as continuous infusion over 8 h intervals)	Until occlusion of the causative aneurysm, or ≤36 h (short-term)	Yes (Calcium antagonists, euvolemia)	Mortality, rebleeding, delayed cerebral ischemia, dydrocephalus, clinical outcome (mRS)	6 months
Post *et al.* 2021/RCT	955 (475/480)	iv. (1 g bolus followed by 1 g infusion every 8 h)	Until surgical treatment or ≤24 h, whichever came first (short-term)	No	Mortality, rebleeding, hydrocephalus, delayed cerebral ischemia, clinical outcome (mRS)	6 months

DVT: Deep venous thrombosis; ER: Emergency; GOS: Glasgow outcome scale; ICU: Intensive care unit; iv.: Intravenous; mRS: Modified rankin scale; RCT: Randomized controlled trial.

All studies were of a considerably high methodological quality as shown in Supplementary File 2. Funnel plots were made for outcomes that had ten or more studies. Symmetrical funnel plots (Supplementary Figures 1 & 2) suggest that there was no small study or publication bias.

### Effect of intervention on outcomes

The outcomes included in the analysis were rebleeding risk, poor clinical outcome, delayed cerebral ischemia and all-cause mortality. Four major subgroups discussed previously were identified and then analyzed for each outcome.

#### Risk of rebleeding (Figure 2)

A total of 12 studies reported the effect of TXA therapy on the risk of rebleeding after SAH (n = 3359; TXA = 1550; control = 1809). The pooled result showed that TXA use significantly reduced the risk of rebleeding (OR: 0.55; 95% CI: 0.40–0.75; p = 0.0002]. The test for subgroup differences showed no statistically significant subgroup effect (p = 0.61), suggesting that the duration of antifibrinolytic administration and medical ischemia prevention does not change the effect of TXA therapy on rebleeding risk when compared with control.

**Figure 2.**
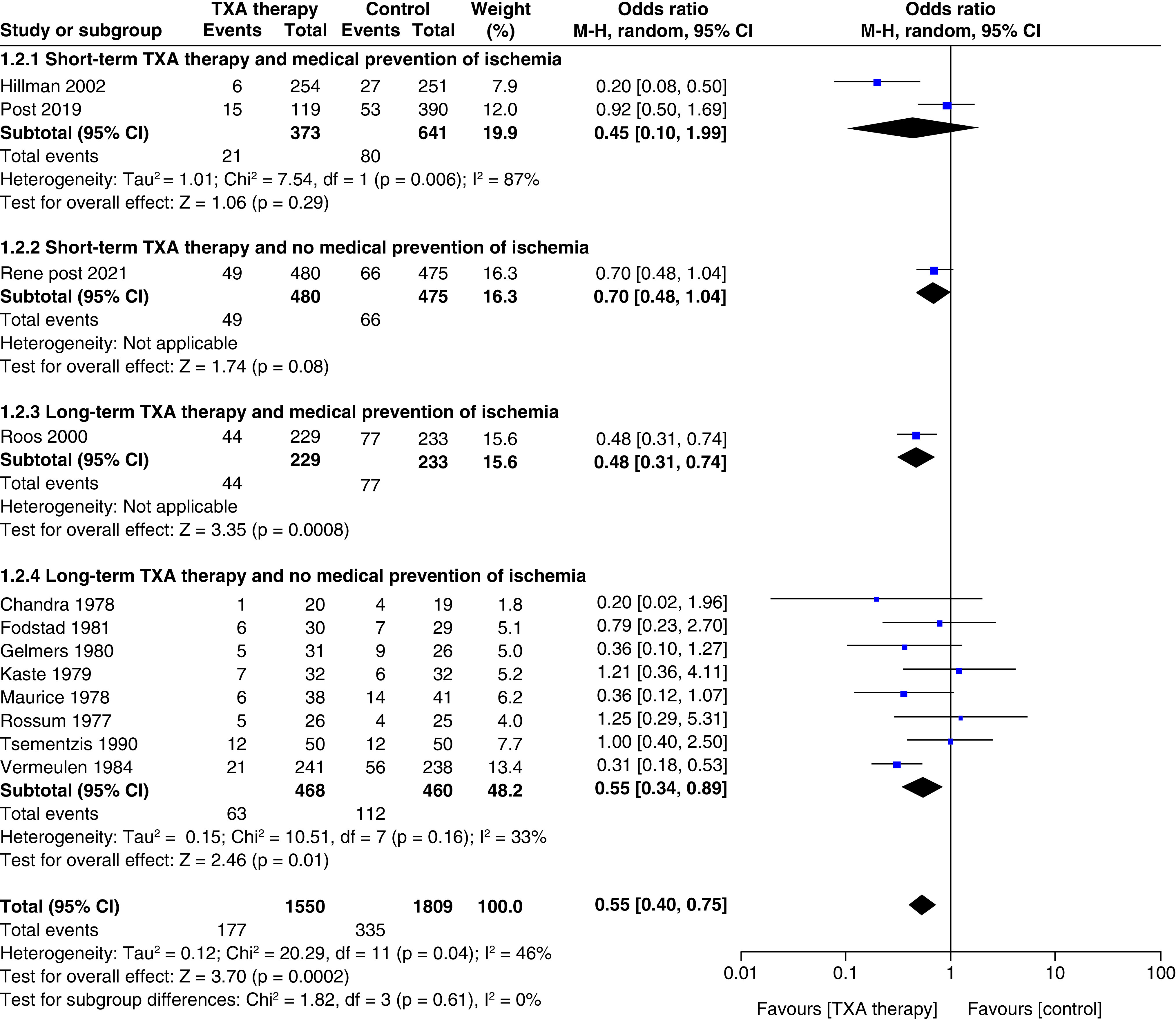
Effect of tranexamic acid therapy on rebleeding risk.

#### Poor clinical outcome (Figure 3)

**Figure 3.**
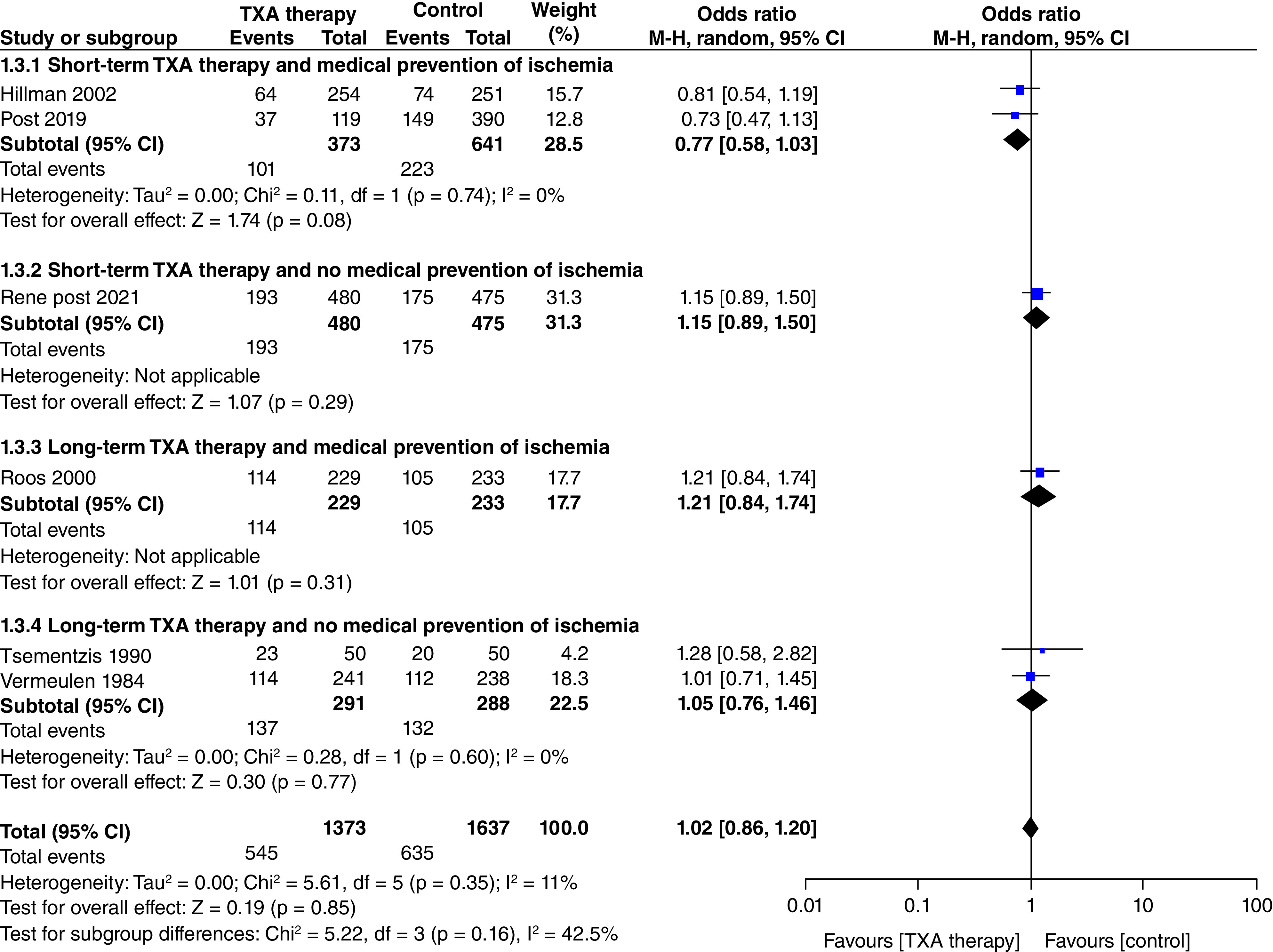
Effect of tranexamic acid therapy on poor clinical outcome.

Six studies presented data on poor clinical outcomes at the end of follow-up (n = 3010; TXA = 1373; control = 1637). There was no significant decrease in poor clinical outcomes with TXA therapy as compared with control (OR: 1.02; 95% CI: 0.86–1.20; p = 0.85). This lack of effect was consistent among all four subgroups as shown in Figure 3

#### Delayed cerebral ischemia (Figure 4)

**Figure 4.**
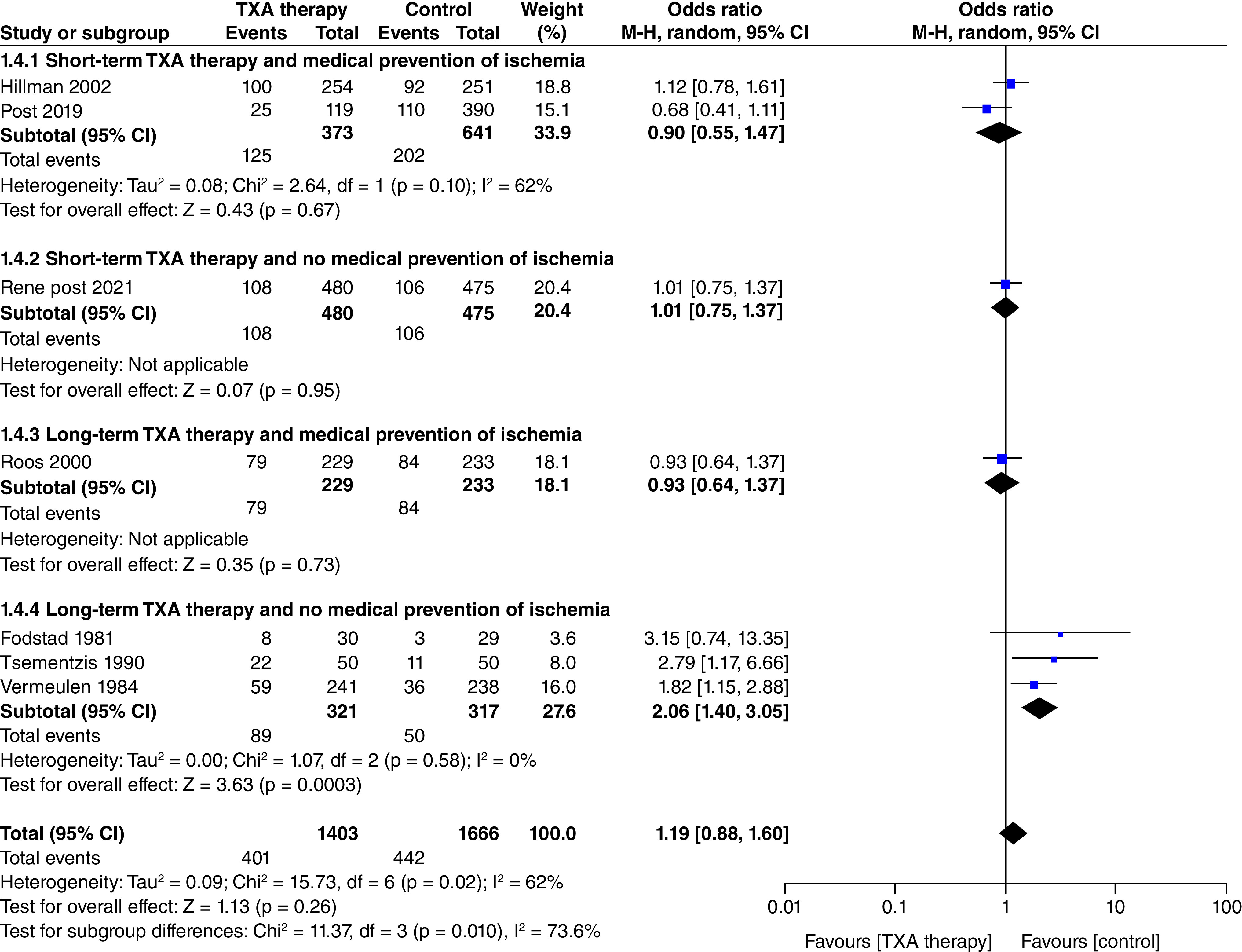
Effect of tranexamic acid therapy on delayed cerebral ischemia.

In the seven studies that reported data on delayed cerebral ischemia (n = 3069; TXA = 1403; control = 1666), TXA treatment did not significantly increase the risk of cerebral ischemia (OR: 1.19; CI: 0.88–1.60; p = 0.26). Considerable heterogeneity (I^2^ = 62%) was found in the analysis of this outcome, but the result of the subgroup analysis explains this heterogeneity. The subgroup ‘Long-term TXA therapy and no medical prevention of ischemia’ showed a significantly increased risk of delayed ischemia (OR: 2.06; CI: 1.40–3.05; p = 0.0003) and the test for subgroup differences was statistically significant (p_interaction_ <0.1) (Figure 4 1.4.4). There was no increase in cerebral ischemia in the other subgroups; short-term TXA and medical prevention of ischemia (OR: 0.90; CI: 0.55–1.47; p = 0.67) (Figure 4 1.4.1), short-term TXA and no medical prevention of ischemia (OR: 1.01; CI: 0.75–1.37; p = 0.95) (Figure 4 1.4.2) and long term TXA and medical prevention of ischemia (OR: 0.93; CI: 0.64–1.37; p = 0.73).

#### All-cause mortality (Figure 5)

Twelve studies reported data on all-cause mortality (n = 3359; TXA = 1550; control = 1809). TXA therapy had no significant effect on all-cause mortality (OR: 0.92; CI: 0.72–1.17; p = 0.50) and similar results were seen in the subgroup analysis. The overall heterogeneity was acceptable (<50%).

**Figure 5.**
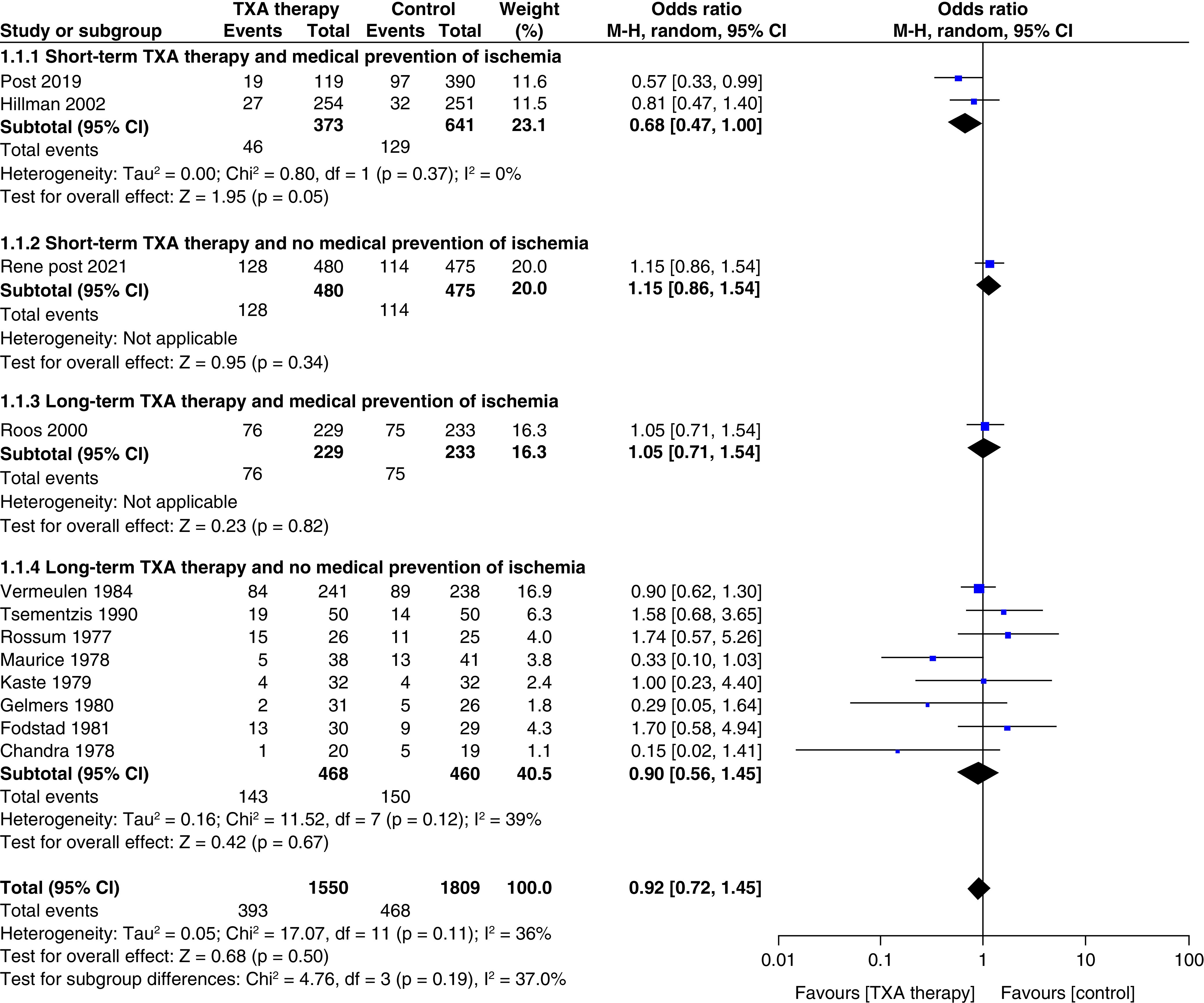
Effect of tranexamic acid therapy on all-cause mortality.

## Discussion

This meta-analysis shows that neither short-term (started at the time of SAH diagnosis and continued <72 h) nor long-term (started at the time of SAH diagnosis and continued >72 h) antifibrinolytic therapies improve clinical outcome in patients with aneurysmal SAH, irrespective of whether or not the anticipated vasospasm (or cerebral ischemia) is medically prevented. Although there is a decrease in the rebleeding risk with long-term TXA therapy, the evidence for an overall efficacy in terms of a better clinical outcome and mortality benefit is lacking. Our findings conform with the previous studies but provide stronger evidence by including a larger pool of data from more recent trials and add significant value by answering some pertinent questions raised in the two previous meta-analyses. Gaberel *et al.* and Baharoglu *et al.* [2,22] suggested that short-term treatment, especially with medical prevention of vasospasm, may decrease rebleeding risk without an increase in the risk of cerebral ischemia, hence leading to improved clinical outcomes. Although our results showed that the risk of delayed cerebral ischemia was not increased with short-term TXA therapy, there was no significant decrease in the rebleeding risk nor an improvement in clinical outcomes.

Rebleeding is the most feared complication after SAH as it carries a high mortality [23]. The role of TXA in clot stabilization and its antifibrinolytic properties initially nurtured the idea of using it in SAH, and the results of various clinical trials supported it as well [24]. In 2003, Roos suggested that although there is a clear benefit in terms of rebleeding risk, there is an associated increase in delayed cerebral ischemia, resulting in an overall inefficacy when it comes to mortality and clinical outcome [24]. Consequently, TXA therapy largely fell out of favor at most neurosurgical centers. In the earlier half of the last decade, it was proposed that short-term TXA therapy (given before the window of cerebral ischemia), especially along with antivasospasm measures like calcium channel blockers and hypervolemic therapy, etc., does not increase the risk of delayed cerebral ischemia in addition to significantly reducing rebleeding. In order to evaluate the credibility of this hypothesis, a large cohort study and the ULTRA trial were commenced, and have recently been completed [19,21]. The importance of newer evidence, especially the ULTRA trial, is paramount because most previous trials were carried out in an era when the practice of early aneurysm obliteration was almost non existent. According to our results, short-term TXA treatment did not significantly decrease the rebleeding risk and did not improve any other outcomes. Long-term treatment, on the other hand, significantly reduced the rebleeding risk, but this also came at the cost of increased cerebral ischemia. A recent review that investigated the role of fibrinolysis following SAH concluded that fibrinolysis does not have a proven role in SAH, and decreased platelet function is potentially the major factor in increasing rebleeding after SAH. Thus, while TXA may have some role in clot stabilization (especially when used long-term), there is little to no evidence to support its use at present. Recent studies have also suggested exploring alternative agents such as desmopressin to prevent rebleeding [23].

Recent guidelines suggest that early surgical or endovascular management of the aneurysm (within 24 h) is the only definitive treatment [25] to prevent rebleeding. The success of these procedures is dependent on the condition and resources of individual hospitals [26]. Multidisciplinary teams are usually needed to decide the preferred technique (microsurgical clipping or endovascular coiling) depending on the patient and aneurysm characteristics [25]. Multiple other factors serve as an obstacle to this management including a delay in referral to the neurosurgical units, lack of logistic support or unsuitable clinical condition of the patients at the time of admission [27]. Therefore, additional measures to improve clinical outcomes (by preventing rebleeding and vasospasm) are now a part of standard care. Early hypertension management to keep systolic blood pressure less than 160 mmHg is the usual norm but it is vital to maintain the balance between hypertension and decreased cerebral perfusion to avoid ischemia [25]. Nimodipine administration has consistently shown positive results in terms of clinical outcome and is therefore used in all patients as soon as they are stable [28]. Antifibrinolytic therapies were also previously given as part of these conservative measures, but given the lack of improvement in clinical outcomes, most guidelines do not suggest any benefit from their routine use [2]. The findings of this meta-analysis corroborate these current guidelines.

Although no increased risk of cerebral ischemia was observed with short-term TXA therapy compared with long-term use, a significant clinical improvement in this subgroup was still lacking. Therefore, other factors that might be responsible for poor outcome or mortality must be explored. A Spanish study highlighted that poor outcome in SAH was more likely to be seen in older (>65 years) age, female gender and patients who were hyperglycemic at the time of admission [29]. Comorbidities such as hypertension and diabetes mellitus lead to poorer outcomes [30] while smoking has been surprisingly shown to positively impact the outcome despite it being a risk factor for SAH [31]. Moreover, other serious complications especially hydrocephalus (both acute and chronic), respiratory and cardiac abnormalities and electrolyte disturbances are also associated with poor prognosis after SAH [32]. The abovementioned factors might play a role in the observed lack of improved clinical outcome despite a reduction in rebleeding. Furthermore, standardization of SAH trials and formulation of some core outcomes (specific to SAH) to be reported in each trial are needed to ensure an adequate comparison between trials and gain further progress in SAH management [33].

### Strengths & limitations

Compared with the previous meta-analyses, the power of this study is enhanced due to the inclusion of the most recent studies with a larger data pool. In order to reduce bias and yield reliable results, only the randomized controlled trials with a clearly reported follow-up period for clinical outcome and observational studies with propensity matched data were included. We believe that this significantly reduced the heterogeneity for all the outcomes except cerebral ischemia which might be accounted for by the variability in its definition and interpretation in different studies. This study adds significant value to the current literature by testing the proposed hypothesis regarding the possible benefit of short-term TXA therapy in SAH, especially with ischemia prevention. However, the authors would also like to acknowledge some limitations. The standard of care and treatment guidelines for aneurysmal SAH have significantly evolved over the last two decades (e.g., hypervolemia which was previously used for medical prevention of ischemia, has largely been replaced by euvolemia and calcium channel blockers now). This change in guidelines also explains the use of hypervolemia in older versus euvolemia in newer studies. Although we included the most recent available evidence in our meta-analysis, it must be noted that many trials addressing this topic are older, so all the results may not be completely applicable in modern practice. Clinical heterogeneity also exists owing to the variation in outcomes definitions, disease severity at baseline, the dose of TXA, and the start and duration of TXA after SAH diagnosis. At last, our literature search spanning four databases and bibliographies of all articles was not exhaustive, so it is possible that we did not find all relevant publications.

## Conclusion

According to current evidence, both short-term and long-term antifibrinolytics cannot be recommended for presumed or proven aneurysmal SAH. Although the risk of rebleeding may be significantly reduced with long-term treatment, however, for reasons that are poorly understood, the evidence for an overall benefit in terms of better clinical outcome and mortality rates is lacking. TXA may have some role in clot stabilization, but fibrinolysis (which TXA primarily affects) has never been definitely proven after SAH, reinforcing that it may be time to explore other avenues besides TXA. It may be important to mention that most literature on this topic is old, so newer trials that are more clinically applicable in this era are needed to enhance the strength of evidence.

**Summary points**

- In patients with subarachnoid hemorrhage (SAH), rebleeding is the most important complication.
- Antifibrinolytics reduce the risk of rebleeding in patients with SAH.
- Antifibrinolytic use does not decrease mortality or poor clinical outcome after SAH.
- Long-term antifibrinolytic use without antivasospasm measures increases rates of cerebral ischemia.

## References

[1] Etminan N, Chang H-S, Hackenberg K Worldwide incidence of aneurysmal subarachnoid hemorrhage according to region, time period, blood pressure, and smoking prevalence in the population: a systematic review and meta-analysis. JAMA Neurol. 76(5), 588–597 (2019). 30659573 10.1001/jamaneurol.2019.0006 PMC6515606

[2] Baharoglu MI, Germans MR, Rinkel GJE Antifibrinolytic therapy for aneurysmal subarachnoid haemorrhage. Cochrane Database Syst. Rev. 2013(8), CD001245 (2013). 10.1002/14651858.CD001245.pub2 PMC8407182 23990381

[3] Nieuwkamp DJ, Setz LE, Algra A, Linn FHH, de Rooij NK, Rinkel GJE. Changes in case fatality of aneurysmal subarachnoid haemorrhage over time, according to age, sex, and region: a meta-analysis. Lancet Neurol. 8(7), 635–642 (2009). 19501022 10.1016/S1474-4422(09)70126-7

[4] Schatlo B, Fung C, Stienen MN Incidence and outcome of aneurysmal subarachnoid hemorrhage: the Swiss study on subarachnoid hemorrhage (Swiss SOS). Stroke 52(1), 344–347 (2021). 33272133 10.1161/STROKEAHA.120.029538

[5] Lord AS, Fernandez L, Schmidt JM Effect of rebleeding on the course and incidence of vasospasm after subarachnoid hemorrhage. Neurology 78(1), 31–37 (2012). 22170890 10.1212/WNL.0b013e31823ed0a4 PMC3466499

[6] Starke RM, Kim GH, Fernandez A Impact of a protocol for acute antifibrinolytic therapy on aneurysm rebleeding after subarachnoid hemorrhage. Stroke 39(9), 2617–2621 (2008). 18658042 10.1161/STROKEAHA.107.506097

[7] Suarez JI. Diagnosis and management of subarachnoid hemorrhage. Contin. Minneap. Minn. 21(5), 1263–1287 (2015). 10.1212/CON.0000000000000217 26426230

[8] Higgins JPT, Altman DG, Gotzsche PC The Cochrane Collaboration's tool for assessing risk of bias in randomised trials. Br. Med. J. 343(2), d5928–d5928 (2011). 22008217 10.1136/bmj.d5928 PMC3196245

[9] Wells G, Shea B, O'Connell D The Newcastle-Ottawa Scale (NOS) for assessing the quality of nonrandomised studies in meta-analyses (2013). http://www.ohri.ca/programs/clinical_epidemiology/oxford.asp

[10] Chandra B. Treatment of subarachnoid hemorrhage from ruptured intracranial aneurysm with tranexamic acid: a double-blind clinical trial. Ann. Neurol. 3(6), 502–504 (1978). 354483 10.1002/ana.410030607

[11] Gelmers HJ. Prevention of recurrence of spontaneous subarachnoid haemorrhage by tranexamic acid. Acta Neurochir. (Wien) 52(1–2), 45–50 (1980). 7376944 10.1007/BF01400945

[12] Hillman J, Fridriksson S, Nilsson O, Yu Z, Saveland H, Jakobsson K-E. Immediate administration of tranexamic acid and reduced incidence of early rebleeding after aneurysmal subarachnoid hemorrhage: a prospective randomized study. J. Neurosurg. 97(4), 771–778 (2002). 12405362 10.3171/jns.2002.97.4.0771

[13] Vermeulen M, Lindsay KW, Murray GD Antifibrinolytic treatment in subarachnoid hemorrhage. N. Engl. J. Med. 311(7), 432–437 (1984). 6379455 10.1056/NEJM198408163110703

[14] van Rossum J, Wintzen AR, Endtz LJ, Schoen JH, de Jonge H. Effect of tranexamic acid on rebleeding after subarachnoid hemorrhage: a double-blind controlled clinical trial. Ann. Neurol. 2(3), 238–242 (1977). 10.1002/ana.410020310 365072

[15] Maurice-Williams RS. Prolonged antifibrinolysis: an effective non-surgical treatment for ruptured intracranial aneurysms? Br. Med. J. 1(6118), 945–947 (1978). 346153 10.1136/bmj.1.6118.945 PMC1603860

[16] Roos Y. Antifibrinolytic treatment in subarachnoid hemorrhage: a randomized placebo-controlled trial. STAR Study Group. Neurology 54(1), 77–82 (2000). 10636129 10.1212/wnl.54.1.77

[17] Kaste M, Ramsay M. Tranexamic acid in subarachnoid hemorrhage. A double-blind study. Stroke 10(5), 519–522 (1979). 505491 10.1161/01.str.10.5.519

[18] Tsementzis SA, Hitchcock ER, Meyer CH. Benefits and risks of antifibrinolytic therapy in the management of ruptured intracranial aneurysms. A double-blind placebo-controlled study. Acta Neurochir. (Wien) 102(1–2), 1–10 (1990). 2407050 10.1007/BF01402177

[19] Post R, Germans MR, Tjerkstra MA Ultra-early tranexamic acid after subarachnoid haemorrhage (ULTRA): a randomised controlled trial. Lancet 397(10269), 112–118 (2021). 33357465 10.1016/S0140-6736(20)32518-6

[20] Fodstad H, Forssell A, Liliequist B, Schannong M. Antifibrinolysis with tranexamic acid in aneurysmal subarachnoid hemorrhage: a consecutive controlled clinical trial. Neurosurgery 8(2), 158–165 (1981). 7010203 10.1227/00006123-198102000-00004

[21] Post R, Germans MR, Boogaarts HD Short-term tranexamic acid treatment reduces in-hospital mortality in aneurysmal sub-arachnoid hemorrhage: a multicenter comparison study. PloS ONE 14(2), e0211868 (2019). 30730957 10.1371/journal.pone.0211868 PMC6366882

[22] Gaberel T, Magheru C, Emery E, Derlon J-M. Antifibrinolytic therapy in the management of aneurismal subarachnoid hemorrhage revisited. A meta-analysis. Acta Neurochir. (Wien) 154(1), 1–9 (2012). 22002504 10.1007/s00701-011-1179-y

[23] Hvas CL, Hvas AM. Hemostasis and fibrinolysis following aneurysmal subarachnoid hemorrhage: a systematic review on additional knowledge from dynamic assays and potential treatment targets. Semin. Thromb. Hemost. 48(03), 356–381 (2022). 34261149 10.1055/s-0041-1730346

[24] Roos YBWEM, Rinkel GJE, Vermeulen M, Algra A, van Gijn J. Antifibrinolytic therapy for aneurysmal subarachnoid hemorrhage. Cochrane Database Syst. Rev. (2), CD001245 (2003). 10.1002/14651858.CD001245 12804399

[25] Connolly ES, Rabinstein AA, Carhuapoma JR Guidelines for the management of aneurysmal subarachnoid hemorrhage: a guideline for healthcare professionals from the American Heart Association/American Stroke Association. Stroke 43(6), 1711–1737 (2012). 22556195 10.1161/STR.0b013e3182587839

[26] Zipfel GJ. Editorial: ultra-early surgery for aneurysmal subarachnoid hemorrhage. J. Neurosurg. 122(2), 381–382 (2015). 25403773 10.3171/2014.8.JNS141613

[27] Roos YB, Beenen LF, Groen RJ Timing of surgery in patients with aneurysmal subarachnoid haemorrhage: rebleeding is still the major cause of poor outcome in neurosurgical units that aim at early surgery. J. Neurol. Neurosurg. Psychiatry 63, 490–493 (1997). 9343129 10.1136/jnnp.63.4.490 PMC2169792

[28] Allen GS, Ahn HS, Preziosi TJ Cerebral arterial spasm--a controlled trial of nimodipine in patients with subarachnoid hemorrhage. N. Engl. J. Med. 308(11), 619–624 (1983). 6338383 10.1056/NEJM198303173081103

[29] Rodríguez DR, Matamoros CS, Cúe LF, Hernández JLM, Sánchez YP, Nellar JP. Factors associated with poor outcome for aneurysmal subarachnoid haemorrhage in a series of 334 patients. Neurol. Barc. Spain 32(1), 15–21 (2017). 10.1016/j.nrl.2014.12.006 25704984

[30] Avdagic SS, Brkic H, Avdagic H, Smajic J, Hodzic S. Impact of comorbidity on early outcome of patients with subarachnoid hemorrhage caused by cerebral aneurysm rupture. Med. Arch. 69(5), 280–283 (2015). 26622076 10.5455/medarh.2015.69.280-283 PMC4639362

[31] Slettebø H, Karic T, Sorteberg A. Impact of smoking on course and outcome of aneurysmal subarachnoid hemorrhage. Acta Neurochir. (Wien) 162(12), 3117–3128 (2020). 32728905 10.1007/s00701-020-04506-3 PMC7593300

[32] Danière F, Gascou G, Menjot de Champfleur N Complications and follow up of subarachnoid hemorrhages. Diagn. Interv. Imaging 96(7–8), 677–686 (2015). 26119863 10.1016/j.diii.2015.05.006

[33] Andersen CR, Presseau J, Saigle V, Etminan N, Vergouwen MDI, English SW. Core outcomes for subarachnoid haemorrhage. Lancet Neurol. 18(12), 1075–1076 (2019). 10.1016/S1474-4422(19)30412-0 31701889

