# Supplementary material for: The efficacy of antifibrinolytic therapy in aneurysmal subarachnoid hemorrhage: a systematic review and meta-analysis
Source: Future Sci OA. 2023 May 16;9(6):FSO866. doi: 10.2144/fsoa-2023-0014 (PMC10203907; doi:10.2144/fsoa-2023-0014)
Supplement: Supplementary file 1 [file fsoa-09-866-s1.docx]

***
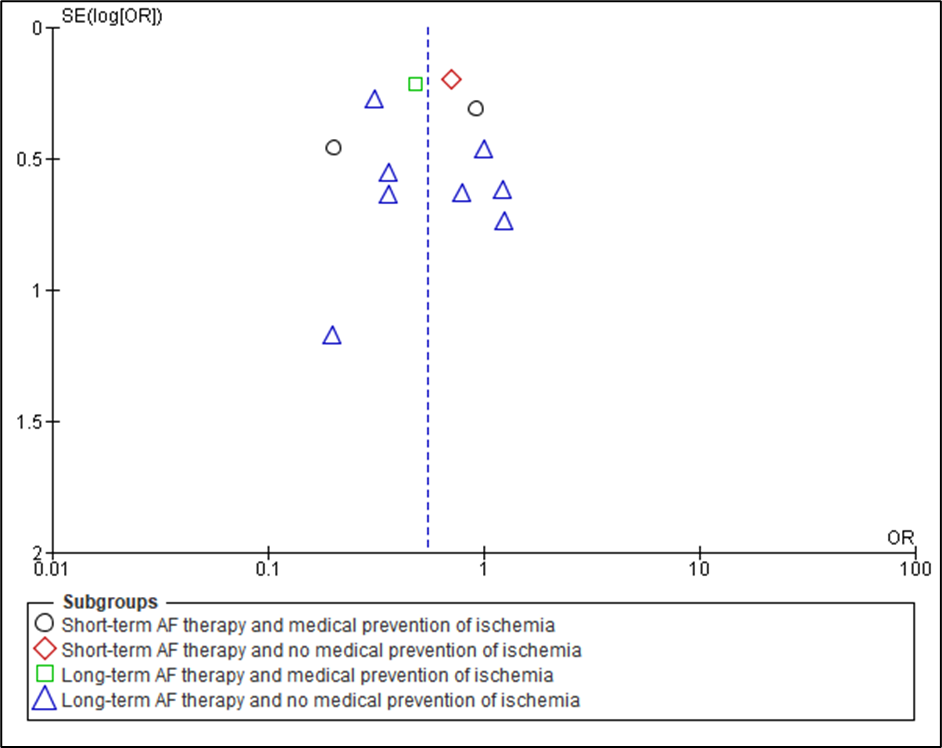
***

Supplementary Figure 1: Funnel plot for the effect of AF therapy on rebleeding risk


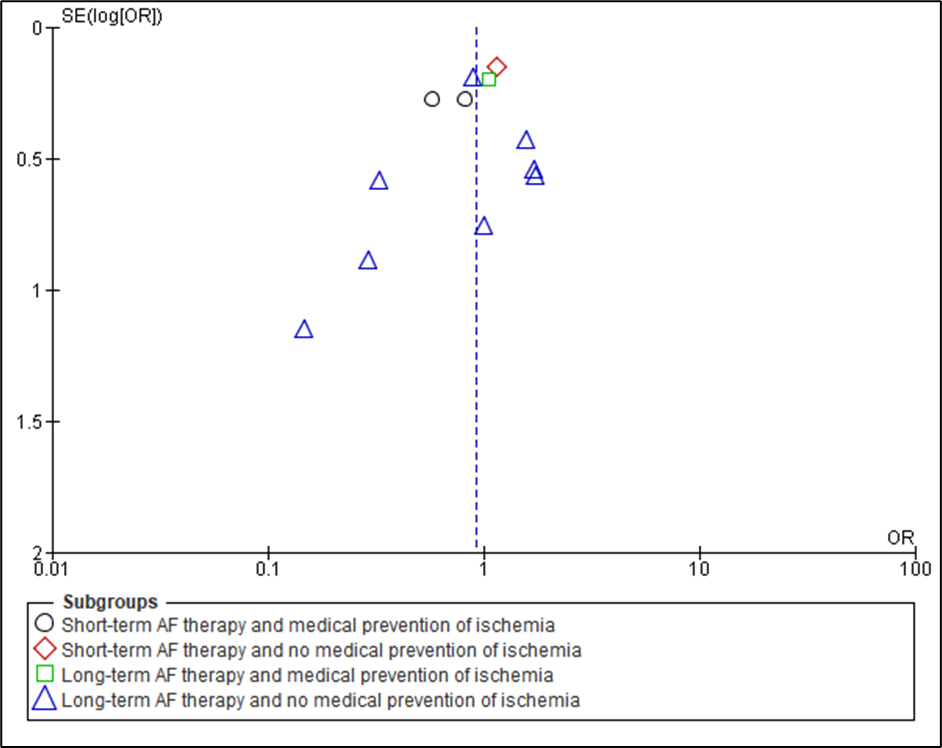


Supplementary Figure 2: Funnel plot for the effect of AF therapy on all-cause mortality
