# Supplementary material for: The efficacy of antifibrinolytic therapy in aneurysmal subarachnoid hemorrhage: a systematic review and meta-analysis
Source: Future Sci OA. 2023 May 16;9(6):FSO866. doi: 10.2144/fsoa-2023-0014 (PMC10203907; doi:10.2144/fsoa-2023-0014)
Supplement: Supplementary file 2 [file fsoa-09-866-s2.docx]

**Date:** April 14, 2023

**PUBMED** 277

("Subarachnoid Hemorrhage"[MeSH Terms] OR "aneurysmal subarachnoid hemorrhage"[All Fields]) AND ("Antifibrinolytic Agents"[MeSH Terms] OR "Tranexamic Acid"[MeSH Terms] OR "Aminocaproic Acid"[All Fields] OR "antifibrinolytics"[All Fields])

**SCIENCE DIRECT** 315

("Subarachnoid Hemorrhage") AND ("Antifibrinolytic Agents")

**Google Scholar** 915

("Subarachnoid Hemorrhage") AND ("Antifibrinolytic Agents")

**COCHRANE** 37

Search Name:

Date Run: 14/04/2023 17:02:56

Comment:

ID Search Hits

#1 MeSH descriptor: [Subarachnoid Hemorrhage] explode all trees 824

#2 aneurysmal subarachnoid hemorrhage (Word variations have been searched) 1395

#3 MeSH descriptor: [Antifibrinolytic Agents] explode all trees 1040

#4 MeSH descriptor: [Tranexamic Acid] explode all trees 1500

#5 Aminocaproic Acid (Word variations have been searched) 328

#6 antifibrinolytics (Word variations have been searched) 1575

#7 #1 OR #2 1334

#8 #3 OR #4 OR #5 OR #6 1963

#9 #7 AND #8 37

**PUBMED** 277

**SCIENCE DIRECT** 315

**Google Scholar** 915

**COCHRANE** 37

Total 1544

After duplicates removal 514
