# Supplementary material for: The efficacy of antifibrinolytic therapy in aneurysmal subarachnoid hemorrhage: a systematic review and meta-analysis
Source: Future Sci OA. 2023 May 16;9(6):FSO866. doi: 10.2144/fsoa-2023-0014 (PMC10203907; doi:10.2144/fsoa-2023-0014)
Supplement: Supplementary file 3 [file fsoa-09-866-s3.docx]

**Summary of risk of bias assessment of the included studies**


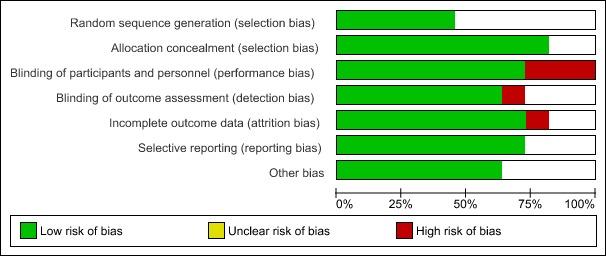


*Risk of bias graph: review authors' judgements about each risk of bias item presented as percentages across all included RCTs.*


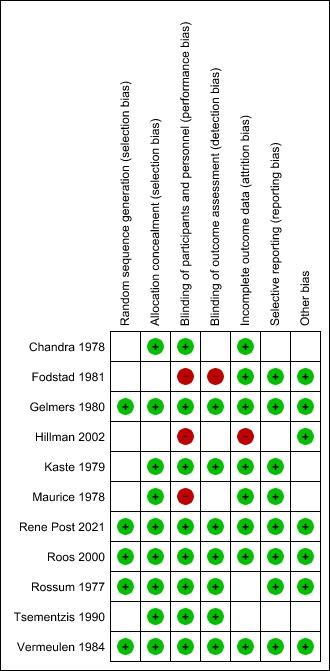


*Risk of bias summary: review authors' judgements about each risk of bias item for each included RCT*

*Newcastle*-*Ottawa Quality Assessment scoring of the included observational cohort study*

| **Study** | **Selection** | **Comparability** | **Outcome** | **Total score** | **Result** |
| --- | --- | --- | --- | --- | --- |
| Post et al | ******** | ***** | ****** | 7 | High-quality Study |
